# Delayed presentation of traumatic giant left anterior descending artery pseudoaneurysm as acute coronary syndrome and cardiogenic shock: a case report

**DOI:** 10.1186/s12872-026-06324-4

**Published:** 2026-07-21

**Authors:** Mustafa Uçar

**Affiliations:** Department of Cardiology, Manisa Celal Bayar University Faculty of Medicine, Manisa, Türkiye

**Keywords:** Coronary pseudoaneurysm, Acute coronary syndrome, Blunt chest trauma, Cardiogenic shock, Left anterior descending artery, Covered stent, Percutaneous coronary intervention

## Abstract

**Background:**

Coronary artery pseudoaneurysms are rare vascular abnormalities that may develop after percutaneous coronary intervention, infection, or trauma. Although many cases remain asymptomatic, pseudoaneurysms may occasionally present with life-threatening complications. Delayed presentation of traumatic coronary pseudoaneurysm as acute coronary syndrome (ACS) complicated by cardiogenic shock is extremely uncommon.

**Case presentation:**

A 74-year-old man presented with chest pain, dyspnea, hypotension, and signs of cardiogenic shock. Electrocardiography demonstrated anterior myocardial infarction. Emergency coronary angiography revealed proximal occlusion of the left anterior descending artery (LAD) associated with a giant pseudoaneurysm and severe circumflex artery stenosis. Given the patient’s hemodynamic instability and high surgical risk related to multiple comorbidities, percutaneous treatment was selected. Following restoration of coronary flow, a covered stent was implanted to seal the pseudoaneurysm. Subsequently, complex bifurcation intervention using the double kissing crush technique was performed. Residual pseudoaneurysm filling was completely eliminated with prolonged balloon inflation. Further history revealed a motor vehicle accident five months before presentation, suggesting a presumed traumatic etiology. The patient recovered uneventfully and was discharged on the fifth day.

**Conclusions:**

This case highlights an unusual delayed presentation of traumatic giant LAD pseudoaneurysm as ACS complicated by cardiogenic shock. Careful clinical history taking may help identify traumatic etiologies. Complex percutaneous coronary intervention with covered stent implantation may represent a feasible treatment option in carefully selected high-risk patients.

**Supplementary Information:**

The online version contains supplementary material available at https://doi.org/10.1186/s12872-026-06324-4.

## Background

Coronary artery aneurysms are uncommon vascular abnormalities defined as localized dilatations exceeding 1.5 times the diameter of the adjacent normal coronary artery. Coronary artery pseudoaneurysms are far less common and result from disruption of the arterial wall with containment of blood by the surrounding connective tissue rather than by the normal vessel wall layers. Unlike true aneurysms, pseudoaneurysms lack one or more layers of the arterial wall and therefore carry a higher risk of rupture, thrombosis, and distal embolization [1, 2]. 

Most coronary pseudoaneurysms occur as complications of percutaneous coronary interventions, coronary artery perforation, or cardiac surgery. Less common etiologies include infection, vasculitis, and blunt or penetrating chest trauma. The clinical presentation is highly variable, ranging from incidental findings to life-threatening complications such as ACS, cardiac tamponade, or sudden cardiac death [3–5]. 

Traumatic coronary artery pseudoaneurysms are particularly rare and may present weeks to months after the initial injury, making diagnosis challenging. Delayed presentation as ACS complicated by cardiogenic shock has been reported only sporadically in the literature [6–8]. 

Herein, I report a case of a giant LAD pseudoaneurysm, likely secondary to prior blunt chest trauma, presenting as ACS and cardiogenic shock five months after a motor vehicle accident. The patient was successfully managed with complex percutaneous coronary intervention involving covered stent implantation followed by double kissing crush bifurcation stenting.

## Case report

A 74-year-old man presented to the emergency department with chest pain and dyspnea. The patient had no history of Kawasaki disease, systemic vasculitis, connective tissue disorders, previous coronary intervention, cardiac surgery, infective endocarditis, or other known conditions associated with coronary artery aneurysm or pseudoaneurysm formation.

**Fig. 1:**
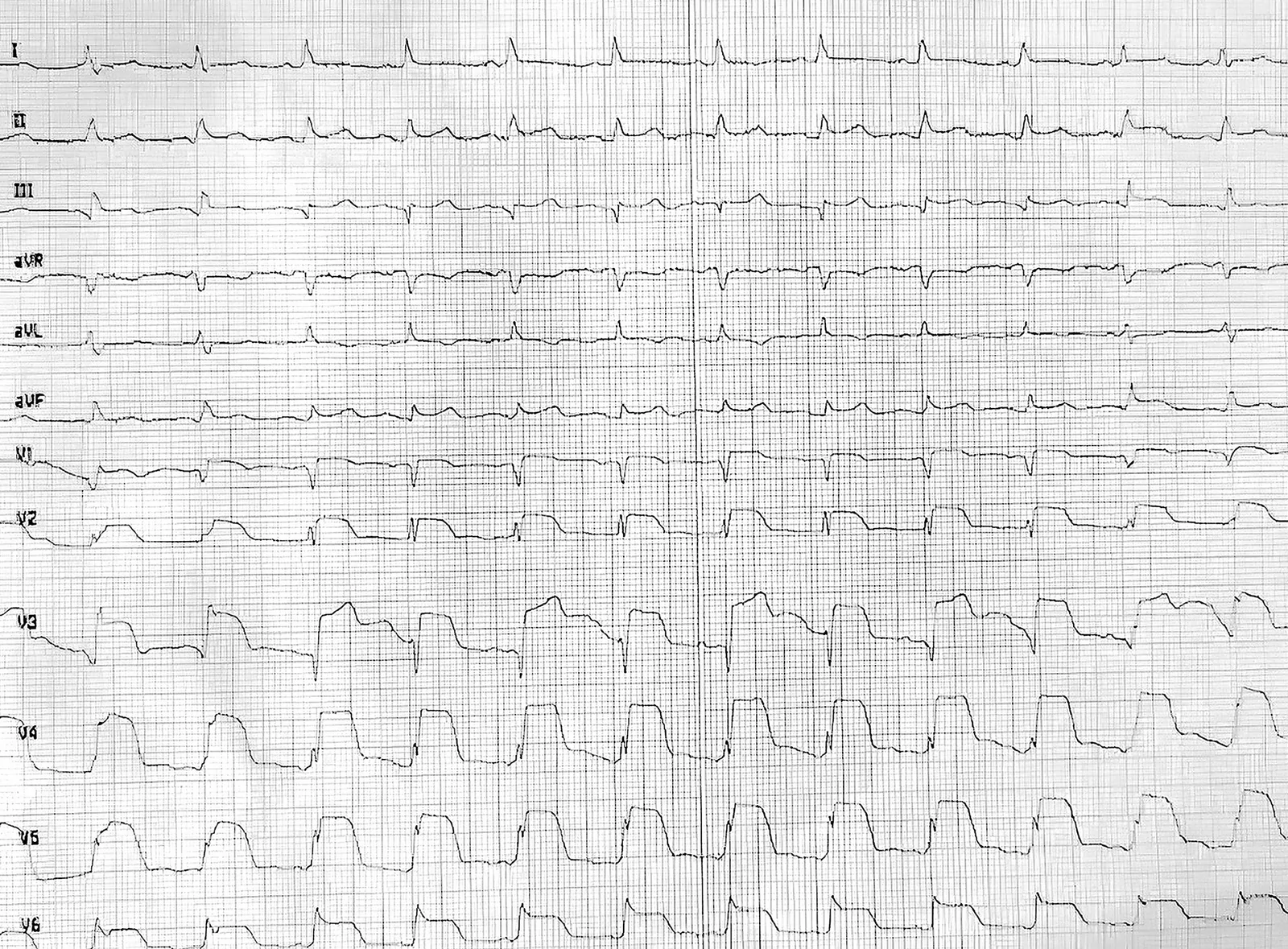
Initial electrocardiogram obtained at presentation demonstrating anterior ST-segment elevation myocardial infarction with ST-segment elevation in the anterior precordial leads

**Fig. 2:**
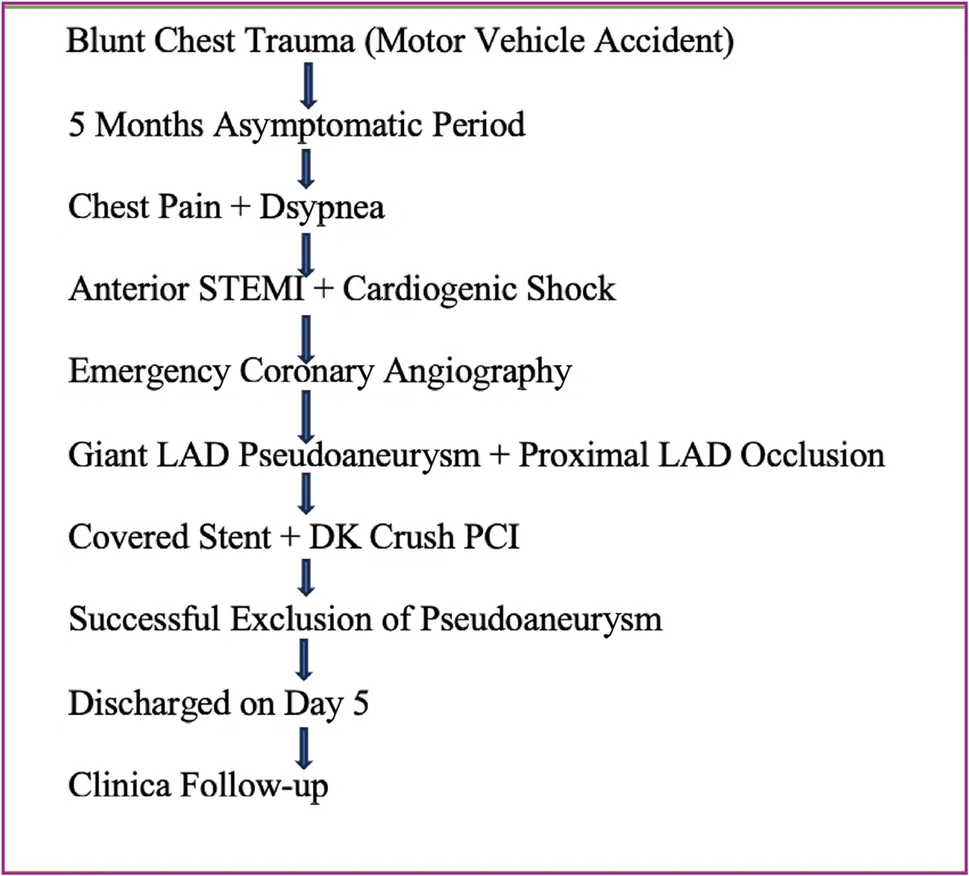
Timeline of the patient’s clinical course from blunt chest trauma to presentation with acute coronary syndrome, emergency percutaneous coronary intervention, and hospital discharge

Physical examination revealed bilateral pulmonary rales, hypotension (80/50 mmHg), and tachycardia (heart rate: 124 beats/min) consistent with cardiogenic shock. Electrocardiography demonstrated anterior ST-segment elevation myocardial infarction (Fig. 1). The overall clinical course of the patient is summarized in Fig. 2.

**Fig. 3:**
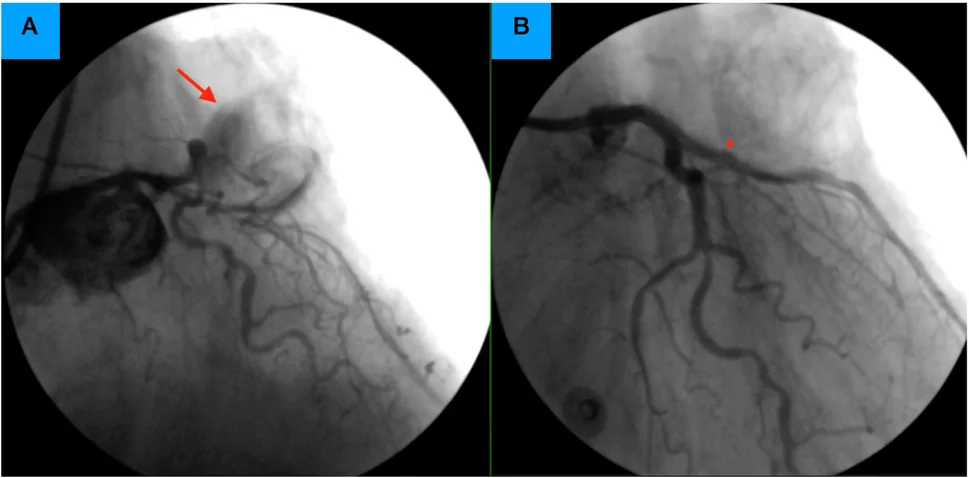
Coronary angiography demonstrating the culprit lesion. **A** Initial coronary angiography demonstrating a giant pseudoaneurysm arising from the proximal LAD immediately proximal to the culprit occlusive lesion (red arrow), together with severe ostial circumflex stenosis. **B** After restoration of coronary flow and covered stent implantation, residual contrast filling of the pseudoaneurysm (red arrowhead) is visible before prolonged balloon inflation

Emergency coronary angiography revealed a giant pseudoaneurysm arising from the proximal LAD, immediately proximal to the culprit LAD occlusion, together with severe (90%) ostial circumflex artery stenosis. Following initial balloon dilatation, restoration of coronary flow allowed clearer visualization of the pseudoaneurysm, its neck, and the culprit lesion (Fig. 3A, Supplementary Video 1).

Given the patient’s hemodynamic instability and significant comorbidities, including chronic obstructive pulmonary disease and cachexia, a percutaneous treatment strategy was selected. Subsequently, a 3.5 × 19 mm Graftmaster covered stent was successfully implanted across the pseudoaneurysm neck, resulting in immediate hemodynamic improvement.

**Fig. 4:**
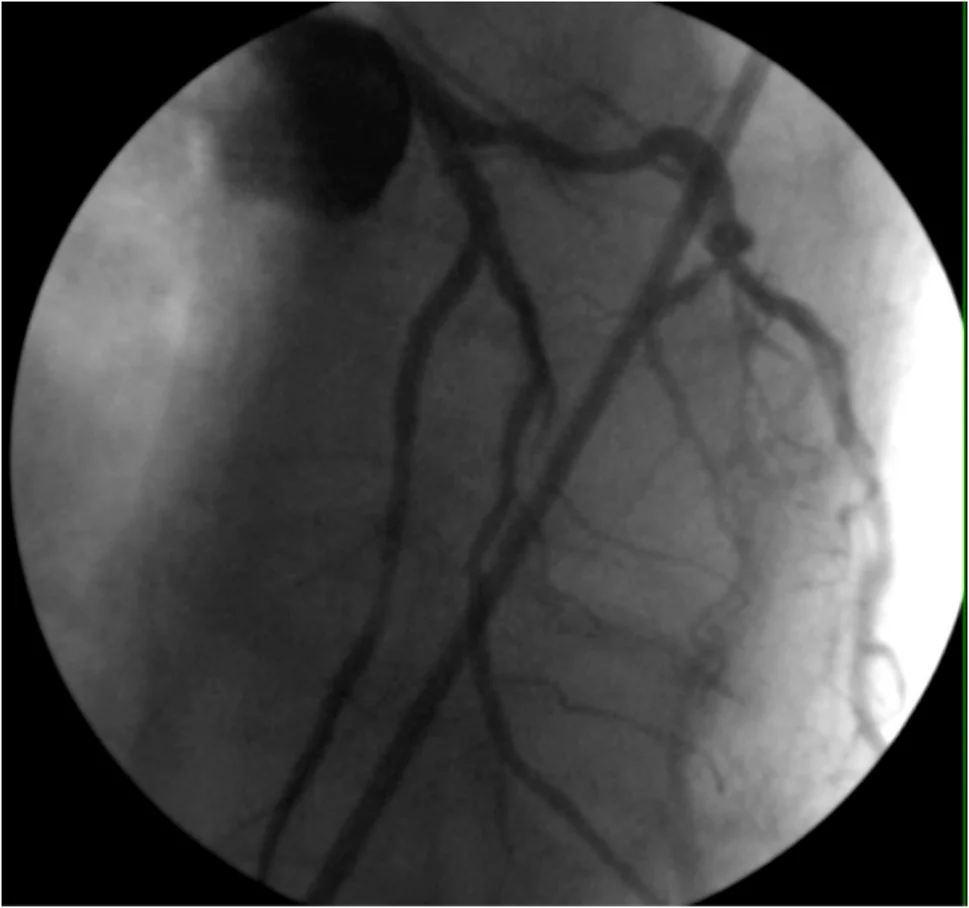
Final angiographic result after covered stent implantation, prolonged balloon inflation, and DK crush bifurcation PCI, demonstrating complete exclusion of the pseudoaneurysm with TIMI 3 flow in the LAD and satisfactory bifurcation reconstruction

Because of the complex LAD-circumflex bifurcation lesion, a double kissing crush bifurcation stenting technique was performed. Residual filling of the pseudoaneurysm was observed after covered stent implantation (Fig. 3B). Therefore, post-dilatation was performed using a 3.5 × 15 mm non-compliant balloon with 90-second inflation, resulting in complete elimination of residual contrast filling and optimal sealing of the pseudoaneurysm neck. Final angiography confirmed complete exclusion of the pseudoaneurysm and restoration of coronary flow (Fig. 4).

Further history obtained after stabilization revealed a motor vehicle accident that had occurred five months before presentation, suggesting a presumed traumatic etiology. The patient was discharged on the fifth day with dual antiplatelet therapy consisting of aspirin and ticagrelor. At the 6-month clinical follow-up, he remained asymptomatic without recurrent chest pain or other ischemic symptoms. Follow-up coronary angiography or coronary computed tomography angiography was not performed.

## Discussion

Coronary artery aneurysms are uncommon vascular abnormalities that can be classified as true aneurysms or pseudoaneurysms. True aneurysms involve all three layers of the arterial wall and are most commonly associated with atherosclerosis, whereas pseudoaneurysms result from disruption of the vessel wall with containment of blood by the surrounding tissues and lack one or more normal arterial wall layers. Because of their structural instability, pseudoaneurysms are associated with a higher risk of rupture, thrombosis, and distal embolization. Their exact incidence remains uncertain because conventional coronary angiography alone may not reliably distinguish pseudoaneurysms from true aneurysms. Most reported coronary pseudoaneurysms occur following percutaneous coronary intervention, coronary perforation, or cardiac surgery, whereas traumatic coronary pseudoaneurysms remain exceptionally rare [1–5]. 

The most notable aspect of the present case is the delayed presentation of a presumed traumatic giant left anterior descending artery pseudoaneurysm as acute coronary syndrome complicated by cardiogenic shock. Blunt chest trauma is a recognized but uncommon cause of coronary artery injury. Clinical manifestations may occur immediately after trauma or remain clinically silent for weeks to months. Consequently, establishing the diagnosis may be challenging unless a careful clinical history is obtained. In our patient, the history of a motor vehicle accident was identified only after successful stabilization, suggesting a delayed evolution of coronary artery wall injury leading to pseudoaneurysm formation. Similar delayed presentations have been reported only sporadically in the literature [6–8]. 

Although the temporal relationship is suggestive of a traumatic origin, the precise mechanism of pseudoaneurysm formation cannot be determined retrospectively. Blunt chest trauma may have caused a small, initially unrecognized coronary artery perforation or an intramural coronary hematoma that progressively evolved into a pseudoaneurysm over subsequent months. Both mechanisms have been described following blunt chest trauma and may explain the delayed clinical presentation observed in this patient [6, 7]. 

Clinical manifestations of coronary pseudoaneurysms are highly variable and range from incidental findings to life-threatening complications including myocardial ischemia, distal embolization, rupture, cardiac tamponade, and sudden cardiac death. Acute coronary syndrome may occur as a consequence of thrombus formation within the pseudoaneurysm sac, distal embolization, or mechanical compromise of coronary blood flow. The presence of cardiogenic shock in our patient further highlights the potentially catastrophic consequences of this condition [4, 5, 9]. 

Intravascular imaging modalities such as intravascular ultrasound (IVUS) or optical coherence tomography (OCT) may provide more accurate characterization of coronary aneurysmal lesions by defining vessel wall integrity and differentiating pseudoaneurysms from true aneurysms. In the present case, intravascular imaging was not performed because of the patient’s hemodynamic instability and the need for immediate reperfusion therapy. Therefore, the diagnosis was based on angiographic findings together with the clinical history and procedural appearance, representing an important limitation of this report.

In the present case, the most likely mechanism of acute coronary syndrome was thrombus formation within the pseudoaneurysm with subsequent distal thromboembolization, resulting in abrupt LAD occlusion. Progressive in-situ thrombosis within the pseudoaneurysm sac may also have contributed to coronary flow limitation. Although external compression of the coronary lumen by the giant pseudoaneurysm cannot be completely excluded, the angiographic findings and clinical presentation favored a thrombotic mechanism rather than pure mechanical compression [4, 10]. 

Management strategies for coronary pseudoaneurysms remain controversial because of their rarity and the absence of standardized treatment algorithms. Available therapeutic options include surgical repair, coil embolization, covered stent implantation, and conservative management in selected patients. Treatment should be individualized according to pseudoaneurysm location, size, clinical presentation, and patient characteristics [4, 11, 12]. 

Covered stents have become an important percutaneous treatment option for coronary pseudoaneurysms because they provide immediate exclusion of the pseudoaneurysm while preserving vessel patency. However, their use is associated with several limitations, including an increased risk of early stent thrombosis and in-stent restenosis related to delayed endothelialization and the relatively bulky stent structure. Therefore, careful patient selection, meticulous implantation technique, and prolonged dual antiplatelet therapy are generally recommended to minimize these risks [13, 14].

Prolonged balloon inflation following covered stent implantation may optimize stent expansion and apposition, facilitate complete sealing of the pseudoaneurysm neck, and eliminate residual contrast filling. In the present case, a 3.5 × 15 mm non-compliant balloon was maintained for 90 s, resulting in complete angiographic exclusion of the pseudoaneurysm without procedural complications. Although prolonged balloon inflation may transiently increase myocardial ischemia due to interruption of coronary blood flow, this strategy may be beneficial in carefully selected cases where complete sealing of the pseudoaneurysm is essential.

In the present case, a percutaneous strategy was preferred because the patient presented with acute ST-segment elevation myocardial infarction complicated by cardiogenic shock, requiring immediate reperfusion, while advanced age, cachexia, and chronic obstructive pulmonary disease were considered to confer a high surgical risk. Because severe (90%) ostial circumflex stenosis coexisted with the culprit proximal LAD lesion, preservation of the circumflex artery was considered essential. Therefore, a two-stent bifurcation strategy was selected to achieve complete revascularization while maintaining patency of both vessels. Conservative management was not considered appropriate because of the ongoing myocardial infarction, hemodynamic instability, and the potential risks of persistent coronary occlusion and pseudoaneurysm-related complications. To my knowledge, reports describing successful treatment of a giant LAD pseudoaneurysm presenting as acute coronary syndrome and cardiogenic shock using covered stent implantation combined with the double kissing crush technique are extremely limited.

The absence of follow-up coronary imaging represents a limitation, as complete long-term exclusion of the pseudoaneurysm and covered stent patency could not be objectively confirmed.

This case emphasizes the importance of considering coronary pseudoaneurysm in the differential diagnosis of acute coronary syndrome, particularly in patients with a history of previous chest trauma. Recognition of this rare entity may facilitate timely diagnosis and appropriate treatment, potentially preventing fatal complications.

## Conclusion

Coronary artery pseudoaneurysms are rare causes of acute coronary syndrome and may present months after blunt chest trauma. This case highlights the importance of obtaining a detailed clinical history in patients with unusual coronary lesions and acute ischemic presentations. In selected high-risk patients, percutaneous treatment with covered stent implantation may provide an effective alternative to surgical management. Early recognition and individualized management strategies are essential to optimize outcomes and prevent potentially fatal complications.

## Supplementary Information

Supplementary Material 1. Supplementary Video 1. Coronary angiography obtained after initial balloon dilatation demonstrating a giant pseudoaneurysm originating from the proximal left anterior descending artery (LAD). The pseudoaneurysm neck and contrast filling of the aneurysmal sac are clearly visualized. Supplementary Video 1 is provided as a separate video file.

## Data Availability

All data generated or analyzed during this study are included in this published article and its supplementary information files.

## Abbreviations

DK Crush: Double Kissing Crush
ACS: Acute Coronary Syndrome
COPD: Chronic Obstructive Pulmonary Disease
CX: Circumflex Artery
LAD: Left Anterior Descending Artery
PCI: Percutaneous Coronary Intervention
